# Accommodation-Free Head Mounted Display with Comfortable 3D Perception and an Enlarged Eye-box

**DOI:** 10.34133/2019/9273723

**Published:** 2019-08-25

**Authors:** Pawan K. Shrestha, Matt J. Pryn, Jia Jia, Jhen-Si Chen, Hector Navarro Fructuoso, Atanas Boev, Qing Zhang, Daping Chu

**Affiliations:** ^1^Centre for Photonic Devices and Sensors, University of Cambridge, 9 JJ Thomson Avenue, Cambridge CB3 0FA, UK; ^2^Huawei Technologies Duesseldorf GmbH, European Research Centre, Riesstrasse 25, München 80992, Germany

## Abstract

An accommodation-free displays, also known as Maxwellian displays, keep the displayed image sharp regardless of the viewer's focal distance. However, they typically suffer from a small eye-box and limited effective field of view (FOV) which requires careful alignment before a viewer can see the image. This paper presents a high-quality accommodation-free head mounted display (aHMD) based on pixel beam scanning for direct image forming on retina. It has an enlarged eye-box and FOV for easy viewing by replicating the viewing points with an array of beam splitters. A prototype aHMD is built using this concept, which shows high definition, low colour aberration 3D augmented reality (AR) images with an FOV of 36°. The advantage of the proposed design over other head mounted display (HMD) architectures is that, due to the narrow, collimated pixel beams, the high image quality is unaffected by changes in eye accommodation, and the approach to enlarge the eye-box is scalable. Most importantly, such an aHMD can deliver realistic three-dimensional (3D) viewing perception with no vergence-accommodation conflict (VAC). It is found that viewing the accommodation-free 3D images with the aHMD presented in this work is comfortable for viewers and does not cause the nausea or eyestrain side effects commonly associated with conventional stereoscopic 3D or HMD displays, even for all day use.

## 1. Introduction

Wearable displays that seamlessly blend the real and virtual world have been topics of research, in both academia and industry for decades. Recent high-profile products launched by large companies such as Google, Magic Leap, and Microsoft have sparked further consumer and industry interest. Augmented reality (AR) head mounted displays (HMD) are expected to have a disruptive impact on a diverse range of markets, including education, hospitality, construction, sports, and the military [1, 2]. A stereoscopic 3D effect can be created by the HMD through binocular disparity, where the image displayed to the left and right eyes is varied slightly. However, this leads to 3D perception problems such as vergence-accommodation conflict (VAC) where the ocular focal distance conflicts with the intersection distance of the left and right eyes. VAC causes nausea, dizziness, eyestrain, and inaccurate depth perception. This can be avoided by simulating the accommodation cue as well as binocular disparity to eliminate the conflicting depth cues.

Holographic displays perfectly reconstruct the wavefront of the 3D image [3], but the image quality of these systems is currently poor, with problems such as speckle, system complexity, and a currently unfeasible spatial-temporal bandwidth required for video rate 3D images. The viewing angle of holography based 3D displays also is fundamentally limited by the pixel size of the display, with the state of art around 3.7 *μ*m for a viewing angle of ±4.9° [4]. Accommodation correct displays can also be created using a tunable lens [5–8] to temporally adjust the focus of the display to match the image content or by dividing the image onto a discrete set of focal planes [9–12]. However, the spatial temporal information bandwidth required by these systems is still very high, and the optics bulky.

Alternatively, the accommodation cue may be completely removed, ensuring that there can be no VAC. Such a display is in focus no matter where the user's eyes are converging, and thus the accommodation information from the display always matches the user's vergence cue. A similar concept was first discussed by Maxwell in 1860 [13], and this type of image is frequently called a Maxwellian view. Because the accommodation cue is removed, the spatial-temporal bandwidth of these displays is orders of magnitude smaller than comparable holographic displays which are also free of VAC [14]. Similarly, Maxwellian displays do not require eye-tracking or dynamic lenses to eliminate the accommodation depth cue for always in-focus images, and can deliver 3D and depth perception solely from vergence cue through always-in-focus stereoscopic images without cue conflict. This enables the optics to be very compact and with little computational overhead to render images. An accommodation-free HMD built on these principles is presented in this paper.

A conventional Maxwellian display operates by imaging a source area simultaneously through a lens in the pupil of the eye [13]. The approach pursued in this work instead uses a narrow, collimated pixel beam focused through the pupil of the eye and projected onto the retina to create a raster image there. Individual raster images on each eye retina are needed for 3D and depth perception. The optics of the human eye only has minimal impact on the spot size of individual beams in this case, so each spot and hence the raster image itself are perceived as in focus regardless of the accommodation state of the eye.

**Figure 1:**
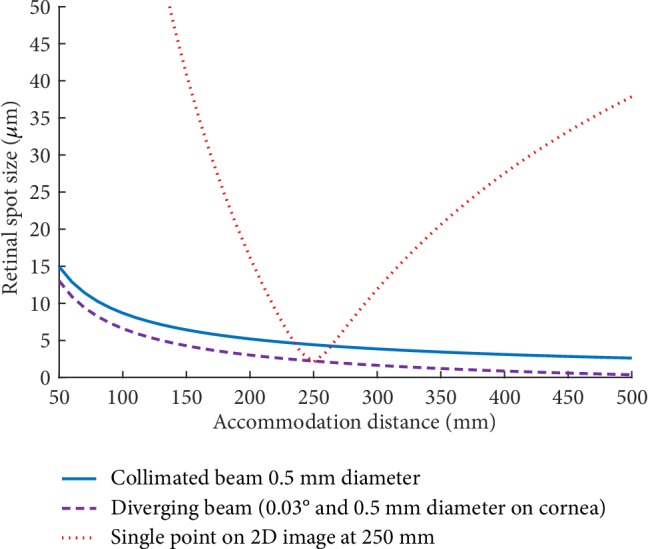
The spot size imaged on the retina for different types of light beam, simulated in Zemax using the Navarro model eye.

A ray tracing simulation was performed to quantify the effect of a Maxwellian display, shown in Figure 1. It can be seen that, for single point on a 2D image at 250 mm (red line), the retinal spot size representing pixel blur increases rapidly for accommodation distances not equal to the object distance. However, the spot size of a 0.5 mm beam representing ideal Maxwellian image pixels of collimated rays (blue line) does not vary significantly, indicating that the image remains in focus over the simulated range. The purple line corresponds to a pixel beam width (0.5 mm on cornea) and divergence (0.03°) matching that of the laser projector used for the prototype developed in this paper, with little retinal spot difference from the ideal Maxwellian condition. The ray tracing simulation was performed in Zemax using the Navarro model eye [15, 16].

The advantage of a Maxwellian display over conventional stereoscopic 3D displays is that VAC can be avoided. VAC is frequently encountered in stereoscopic 3D displays where only the vergence cue is synthesised, and the eyes converge at a distance controlled by the binocular disparity but must focus on the display plane, causing the cue conflict. The visual system expects the cues to match, and headaches and nausea are caused as the user experiences conflicting oculomotor signals to both ciliary muscles controlling focus and the oblique muscles controlling vergence. This conflict can be comfortably tolerated, provided the discrepancy between the accommodation and vergence is small, with a tolerance range approximated by Percival's zone of comfort [17]. For 3D cinema, where the display is reasonably far from the user, the constraints are not a significant limitation. However, for HMD AR this can place severe limitations on the range of depths that can be displayed.

In a Maxwellian display, the image appears in focus regardless of the vergence depth the user is fixating on, ensuring no VAC. In addition, the accommodation of the eye is partially coupled to the vergence distance, causing the eye to naturally adjust focus to match the expected accommodation depth [18]. However, this can cause artefacts where display objects are near each other but with significant depth difference. One object would be expected to be blurred but instead appears in focus and unfused, i.e., double vision. The effects of this are subjectively analysed in the Discussion section. Additional views may be simultaneously projected onto the retina to synthesise the effects of retinal blur [19–21], with between 3 and 26 views demonstrated. However, this increases the required computational load, and either has a significant impact on the refresh rate of the display or requires multiple image generators [22] which is less desirable in a compact HMD.

**Figure 2:**
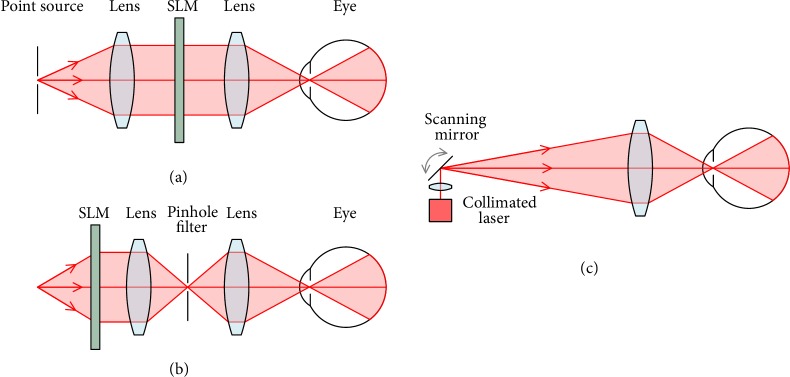
Maxwellian display architecture with (a) collimated illumination, (b) 4f image filtering, and (c) scanning laser projection.

There are three methods to generate a Maxwellian image: collimated illumination, image filtering, and laser projection as depicted in Figure 2. For collimated illumination, a point source is expanded and collimated before illuminating a spatial light modulator (SLM) [19, 23–25]. Similarly, for the filtered image Maxwellian, a light source is used to illuminate an SLM, which is then filtered by a 4f relay through a pinhole [26, 27]. Laser projection Maxwellian display involves creating an image by modulating laser intensity as it is scanned over an angular range by a pair of galvo-mirrors, before the image is collimated through a lens [22, 28]. For all three approaches, a final lens is used to focus the collimated beam to a point through the optics of the eye, to be projected onto the retina. The focal point of the image beam is the exit pupil of the system.

The laser scanning approach is advantageous as no bulky image collimation optics is required, and there is no loss of efficiency at a pinhole filter. Additionally, the beam diameter of the laser scanner may be tuned to optimise the retinal pixel size, whereas the two collimated images have pixel beam diameters defined by the SLM pixel size. The high brightness, contrast, and efficiency of the scanning laser are ideal for a display that must compete with the bright ambient light outdoors but must be battery-powered for portability. Additionally, laser projectors using microelectromechanical system (MEMS) scanning mirrors can be made very compact.

The major challenge of Maxwellian displays is that the collimated image must be focused through the pupil of the eye [29–31]. This requires precise alignment between user and display, causes vignetting as the eyeball rotates, and becomes increasingly challenging as the pupil contracts in bright ambient light.

**Figure 3:**
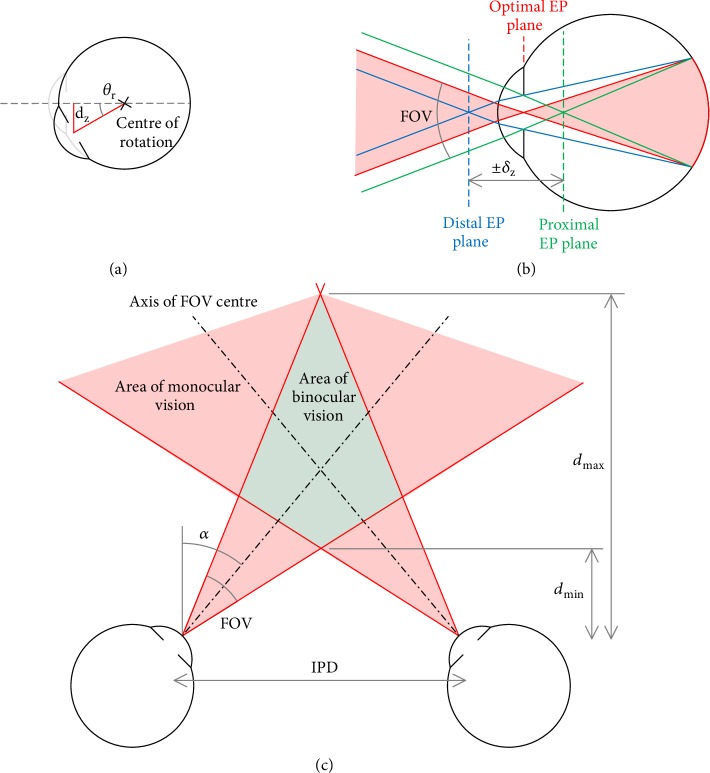
Geometric model of the eye. (a) Ocular pupil position with eyeball rotation. (b) On-axis exit pupil tolerance. (c) The area of binocular vision of a stereoscopic display.

In a perfectly aligned Maxwellian display, the exit pupil falls at the centre of the ocular pupil, allowing half a pupil diameter of movement laterally before vignetting occurs (Figure 3(a)). The lateral displacement *δ*_xy_ of the pupil as the eye rotates can be geometrically calculated using (1). For a standard indoor pupil size of 4 mm [32] a rotation of only 12° is sufficient to cause image vignetting, corresponding to an effective FOV of only 24°:

(1) $$\begin{array}{c} \delta_{xy}=10.2\times \sin\left(\theta_{rot}\right) \end{array}$$

The on-axis alignment tolerance of the display may also be approximated geometrically as the range of positions between the distal and proximal exit pupil planes before the image beam is vignetted by the ocular pupil (Figure 3(b)):

(2) $$\begin{array}{c} \delta_{z}=\pm \frac{p}{2\tan\left(FOV/2\right)} \end{array}$$

The near limit of the region of binocular vision may be modelled geometrically, shown in Figure 3(c). If the centre of the FOV is angled parallel to the optic axis of the eyes (*α* = 0) then the FOV of each eye begins to overlap at a distance of ~100 mm, enabling binocular depth perception in the centre of the FOV:

(3) $$\begin{array}{c} d_{min}=\frac{IPD}{2\tan\left(\alpha +FOV/2\right)} \end{array}$$

where IPD is the interpupillary distance. By increasing the FOV central axis angle, *α*, it is possible to decrease this distance to allow larger objects to be viewed in 3D closer to the eyes. However, larger angles can create a maximum distance for 3D perception if *α* > *FOV*/2:

(4) $$\begin{array}{c} d_{max}=\frac{IPD}{2\tan\left(\alpha -FOV/2\right)} \end{array}$$

An FOV central axis angle of ~5° allows large 3D objects to be perceived close to the eyes without significantly affecting the area of 3D viewing at greater distances.

The eye-box can be enlarged by replicating the exit pupil at different spatial locations. Kim et al. achieve this with a holographic optical element to generate a line of three exit pupils [26]. However, a property of Maxwellian displays is that the perceived position of the image pixels is strongly dependent on the angles of each pixel ray. The holographic exit pupil expanded described above does not preserve pixel ray angles between the three exit pupils, so the image will appear to jump positions when transitioning from one to another and thus cannot be used to increase the permissible eyeball rotation for a larger effective FOV.

Jang et al. extend the eye-box by tracking the user's pupil position and using an additional scanning mirror to reposition the exit pupil [22], but such a system requires eye tracking and additional optics and can only reposition the exit pupil at the display refresh rate.

**Figure 4:**
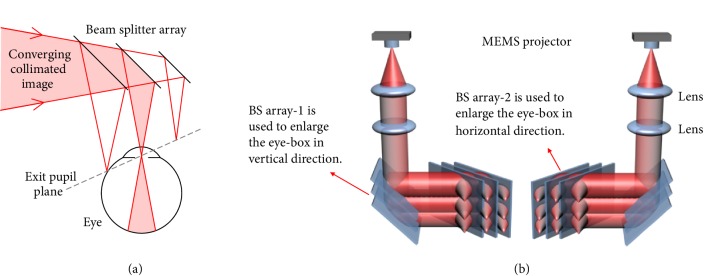
(a) A single beam splitter array to extend the eye-box in one direction. (b) Two sets of beam splitter array may be used to enlarge the eye box in both vertical and horizontal directions by exit pupil duplication.

We propose a simple alternative eye-box enlargement preserving pixel ray alignment between exit pupils without requiring active tracking, by using an array of partially reflective beam splitters to replicate the exit pupil. At each beam splitting surface a fraction of the image beam light is reflected to form an additional exit pupil, with the separation of the viewing points determined by the distance between the surfaces. The disadvantage of such a system is that the exit pupils fall on an inclined plane with respect to the optic axis, shown in Figure 4(a). However, as demonstrated in (2), there is some tolerance in the on-axis exit pupil position. Two sets of beam splitters can create a 2D array of viewing points to enlarge the eye-box in two directions, as shown in Figure 4(b).

## 2. Results

**Figure 5:**
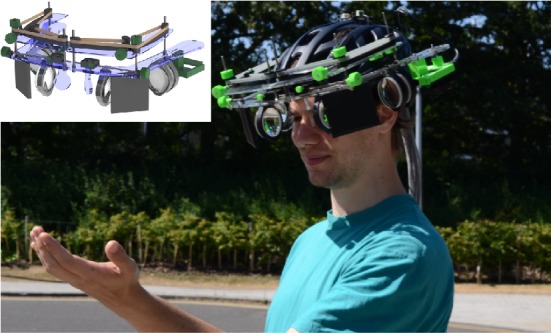
The wearable aHMD with an enlarged eye box. Inset: CAD render of the aHMD design.

A stable head mounted research platform was developed with 5 independent adjustable degrees of freedom per eye to ensure that the display could be used by a wide demographic.  Figure 5 shows a photo of the mechanical system designed for repeatable and stable adjustments of the accommodation-free head mounted display (aHMD) to a wide demographic and prototype by using 3D printing and laser cutting.

**Figure 6:**
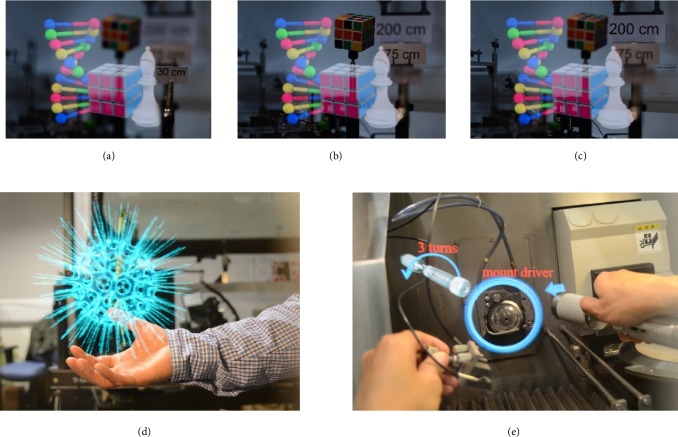
Displayed images as viewed from the aHMD. (a–c) Virtual objects (DNA helix, solved Rubik's cube, and chess piece) always appear in focus as camera is focused on the position marker at 30 cm, 75 cm, and 200 cm. (d) Virtual object of floating virus in air at arm's length (image “Crossview Heliosphaera Radiolaria Polycystina” courtesy of Ramiro Chávez Tovar, Mexico). (e) Application example of AR depicting instructions superimposed over the machine.

Figures 6(a)–6(c) show three images taken by a digital single lens reflection (DSLR) camera through the head mounted prototype with different focus depths, at 30, 75, and 200 cm, respectively. The DNA helix, solved Rubik's cube, and chess piece are projected from the display, whilst the unsolved Rubik's cube and depth markers are arranged in the lab behind the display as a “real-world” scene. It can be seen that as the camera focal length changes, the real-world depth markers come in and out of focus but the aHMD image remains sharp. The minimal colour distortion around the white chess piece demonstrates the achromatic performance of the system with little colour aberration or tinting of the image.  Figure 6(d) shows a virtual object of floating virus displayed in air at arm's length, and Figure 6(e) demonstrates an application example of the AR function depicting instructions superimposed over a machine assembly.

Two supplementary videos [Supplementary-material supplementary-material-1]-[Supplementary-material supplementary-material-1] of the display are also included, both taken with a DSLR camera at 60Hz through the optics of the prototype without modification. The focal length of the camera lens is varied whilst the displayed object remains sharp to demonstrate the accommodation-free properties of the display. A third supplementary [Supplementary-material supplementary-material-1] demonstrates the optical effect when switching between exit pupils, achieved by translating the DSLR. The display optics was unmodified; however, a small aperture was added to the front of the DSLR lens to better simulate a user's ocular pupil; however this significantly affects image quality.

**Figure 7:**
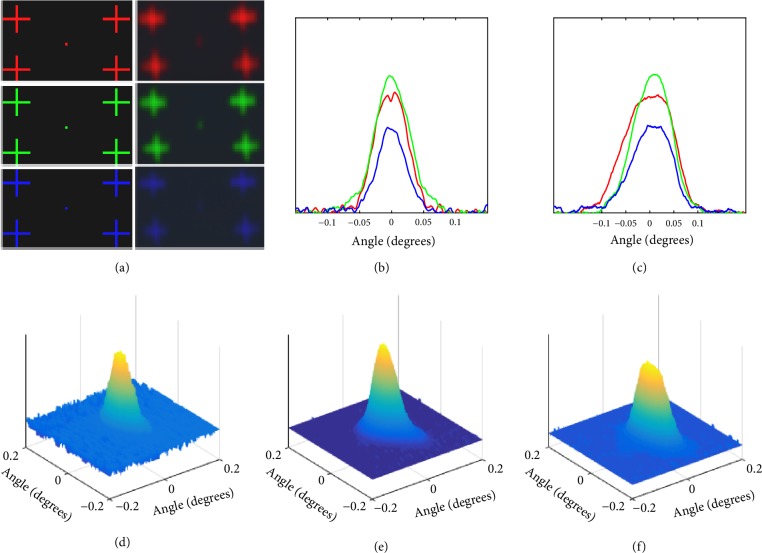
(a) Left: a single pixel of R, G, or B is displayed at the centre of the FOV with four alignment marks in the corners; right: the single pixel R, G, or B pixel with alignment marks is captured on a CCD sensor. (b) PSF measured in the horizontal direction for R, G, and B pixels; (c) PSF measured in vertical direction for R, G, and B pixels; (d–f) direct 3D plot of the PSF for a single R, G, or B pixel, respectively.

The display was objectively analysed with a point-spread function (PSF). To characterise the PSF of the display, a single pixel of red, green, or blue is displayed at the centre of the FOV with four alignment marks in the corners and captured on a charge-coupled device (CCD) sensor (D7000, Nikon) as shown in Figure 7(a). The image diverges as it propagates and is significantly magnified at the CCD sensor placed at a distance of 56 cm, allowing multiple CCD pixels to capture the intensity pattern. The alignment marks allow the captured and displayed images to be scaled and transformed to match, correcting for misalignment in the capture setup.

Output image displayed by the aHMD can be given by,* F*_CG - image_*∗PSF=F*_output_ where* F*_CG - image_ is the spatial distribution of input object (in our case computer generated image) and* F*_output_ is aHMD output image. Here, we record the output image with CCD sensor. Hence,* PSF=PSF*_aHMD_*∗PSF*_CCD_. Here* PSF*_aHMD_ is the point spread function of aHMD and* PSF*_CCD_ is the point spread function of CCD sensor. Each pixel displayed covers >200 pixels of CCD sensor. So, the effect of* PSF*_CCD_ can be neglected.

The Fourier transforms (FT) of both displayed and captured sets of images as shown in Figure 7(a),* FT1* and* FT2*, respectively, are calculated. The PSF of the system may be calculated using

(5) $$\begin{array}{c} PSF={FT}^{-1}\left({FT}_{2}\times {FT}_{1}^{-1}\right) \end{array}$$

The full width at half maximum (FWHM) of the PSF, as depicted in Figures 7(b)-7(c), was measured as 0.03°, 0.03°, and 0.02° for R, G, and B pixels in the horizontal direction and 0.06°, 0.05°, and 0.05° in the vertical direction, respectively.

In addition to the PSF, the spread of a single pixel was measured. A single pixel, as depicted in Figure 7(a) (left), is projected and the corresponding response, as depicted in Figure 7(a) (right), is captured by the CCD sensor. The captured image is scaled until the alignment marks of both images match. Then, the angular spreading of the single pixel is computed as

(6) $$\begin{array}{c} \theta =2\tan^{-1}\left(\frac{np}{2d}\right) \end{array}$$

where* n* is the number of CCD pixels,* p* is the pixel size, and* d* is the distance between CCD and beam splitter. A direct 3D plot of a scaled single pixel (R, G, or B) projected through the system is shown in Figures 7(d)–7(f). The angular spread for R, G, and B in the horizontal direction at FWHM was measured to be 0.03°, 0.03°, and 0.02°and that for vertical direction was measured to be 0.09°, 0.05°, and 0.05°, respectively, similar to the measured PSF values.

A subjective user study was conducted with more than 50 participants comprised of industrial representatives and academic researchers familiar with 3D display technology, with ages ranging from 16 to 60. A range of stereoscopic scenes were presented highlighting image sharpness, chromatic performance, and 3D perception created by the aHMD, with a range of scene depths from 10 cm to 10 m. Questions asked included the following: does this look 3D, and how far away does that virtual object look; can you point how far the object is; how does the image quality look; can you see any chromatic aberration or ghost images; can you see any pixelation; can you read the operating system UI text; does this make you feel dizzy or strain your eyes; is this comfortable to view; can you see any double images if you focus at another depth? All the participants reported the 3D effect to be very convincing for objects from 20 cm to 10 m and pointed to the correct distance when a virtual object was shown at 1 m. The image quality received comments of vivid colour; high contrast; no observation of apparent chromatic aberration, ghost images, or visible image pixellation; and the displayed images and videos blended well with realistic feeling in the surrounding environment. Every participant could read the UI text even without prescription glasses that they normally wear. None of them reported any eyestrain or nausea, and all of them enjoyed the images and videos displayed with comfort. When several objects at different depths were displayed simultaneously, focused double vision was expected; however the effect was neither distracting nor did it affect the ocular comfort of the user. Almost all of users were unaware of the effect until it was brought to their attention. We suspect that, during normal use, users fixate on the region of interest, ensuring that the rest of the image is only perceived as unimportant and so focused double vision is less critical. Additionally, users were encouraged to walk around to explore the utility of the system in a more realistic scenario. Other factors, such as display comfort and stability, were also evaluated with questions such as is this display comfortable on your head and did the image remain visible for the duration of the demonstration. It was found that the current prototype is too heavy for extended use and the image could slip out of the view sometimes due to the shift of the helmet under the weight during prolonged viewing, which was expected and further research is progressing on miniaturising the prototype to a glasses-based format.

The refresh rate of the projector is 60 Hz, enabling high definition (HD) videos of 720p for each eye updating at 60 fps (2×720p60). Previous work from Jang et al. demonstrated a comparable resolution HMD with a time multiplexed accommodation synthesis but a reduced framerate of 10 Hz [22].

## 3. Discussion

High image quality of the designed aHMD was both experimentally and subjectively verified. The images and videos showed bright colours and high contrasts with no observable pixels. The images used by the display to create 3D depths are simple stereoscopic image pairs, computer-generated by rendering the same scene from a slightly altered camera position. Stereo cameras could also be used to record real-world 3D scenes that could be displayed on the aHMD without further processing.

At the same time, 3D viewing perception to eyes by the aHMD through the vergence depth cue was also confirmed. The designed aHMD has been used by more than 50 viewers of a wide range background from administrative staff to experienced game designers. All of them felt comfortable and natural when viewing the displayed 3D virtual objects and none of them reported any nausea or dizziness, even after prolonged periods of usage over a few hours or even all day. It is understood that the difference between the actual image depth and its convergence depth creates VAC which causes nausea, as the eye muscles try to focus on the actual image depth for sharp images while the brain could not reconcile it with the signal to adjust the eye muscles for the corresponding vergence depth at the same time. For the images displayed by the aHMD as proposed in this work, it does not require the eye muscles to adjust the eye focus for the corresponding accommodation depth cue (as in the cases of conventional stereoscopic 3D or HMD displays) because the image is always in focus. This avoids the conflict between these two cues and hence avoids causing nausea and eyestrain. It allows comfortable 3D viewing throughout the depth range, not just in distance. For clarification, the user comments including those by who felt dizzy very quickly to all kinds of existing AR/VR HMDs are qualitative and for demonstrating the effect of the physical system developed here only.

An array of two beam splitters was demonstrated to prove the eye-box extension concept. This can be scaled up easily or replaced by other eye-box extension designs. For the prototype unit, a viewpoint separation of 4 mm was selected for optimal performance, corresponding to a normal pupil diameter of ~4 mm. When the exit pupil spacing matches the ocular pupil diameter no artefacts are seen when transitioning between exit pupils. Greater separation caused dark bands to appear in the image as the pupil moved between viewpoints which is demonstrated in Supplementary [Supplementary-material supplementary-material-1], whilst narrower spacing allows multiple exit pupils to be seen at once. In practice, however, because the exit pupil was not in the plane of the ocular pupil, narrower spacing only caused a small amount of image overlap at the edge, and it was found that it was not particularly apparent. During the user study, the artefacts had to be looked for to be perceived. For a consistent ambient illumination level, such as indoor use, the demonstrated static beam splitter separation works fine and is likely to be sufficient for many use cases including training, CAD development, hospitality, and data manipulation. For environments with greater variation in illumination, such as outdoor sport, defence applications, and construction, it is likely that the solution will encounter problems as the pupil size varies significantly. To an extent this can be mitigated by digitally varying the size of the displayed image provided the exit pupil of the display is not in the plane of the ocular pupil, at the expense of FOV. Dynamic exit pupil spacing could be implemented to eliminate artefacts when transitioning between exit pupils, and this is an area of active further research.

## 4. Materials and Methods

As the proof of concept in the prototype, an acrylic plate was used as the beam splitter array, with the front and back surfaces used to create two exit pupils. A thickness of 6 mm was selected to provide a viewpoint separation of ~4 mm. This was placed directly in front of the eyes, also acting as beam combiner to enable the “real-world” and display to be simultaneously viewed.

A MicroVision MEMS laser projector was selected for the image engine, with dimensions 36x6x53 mm. The laser beam created by the projector is designed to diverge proportionally with the image size [33] specified as 0.03° with a 0.5 mm minimum beam diameter [34].

A neutral density filter was also used to reduce the optical power by three orders of magnitude, and the low reflection efficiency (~4%) of the uncoated acrylic surfaces ensured that the optical power delivered to the eye was much less than the maximum permissible exposure. The projector has a built-in electronic fail safe switch to turn the laser off in the event of MEMS failure to prevent the retinal damage. The spectrum of the projector was tested to contain only the specified 451 nm, 531 nm, 648 nm and wavelengths, without any damaging UV or IR power.

**Figure 8:**
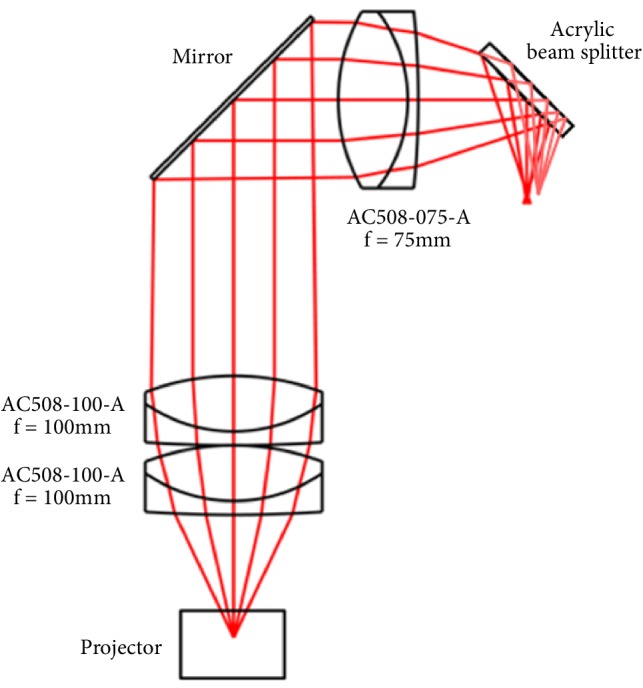
Zemax model of the optical design.

The field of view (FOV) of an accommodation-free display is limited by the focal length of the final lens in the optical train and the collimated image size at the lens given by

(7) $$\begin{array}{c} FOV=2\tan^{-1}\left(\frac{D}{2f}\right) \end{array}$$

where* D* is the lens diameter and* f* is the focal length. A focal length of 75 mm with a 50 mm aperture was decided as a compromise between a diagonal FOV of 36.5° and the proximity of the lens to the user's eye which limits peripheral vision. For comparison the HoloLens by Microsoft has a FOV of ~35°. To maximise image quality achromatic doublet lenses were used in the design and the complete optical train is included in Figure 8. From the projector the laser beam is slightly divergent, but the image beam is very divergent. The image beam is collimated by the first lenses, but this causes the laser beam to become convergent. By placing the second lens 2f from the first, the laser beam can be made collimated for a sharp retinal image, despite the image beam becoming convergent.

## Supplementary Materials

Supplementary Materials Video S1: Accommodation-free virtual virus object. Video S2: Accommodation-free rotating virtual DNA object with changing camera focus. Video S3: Exit pupil transition with virtual virus object.
